# Naloxone administration by law enforcement officers in New York State (2015–2020)

**DOI:** 10.1186/s12954-022-00682-w

**Published:** 2022-09-19

**Authors:** Elham Pourtaher, Emily R. Payne, Nicole Fera, Kirsten Rowe, Shu-Yin John Leung, Sharon Stancliff, Mark Hammer, Joshua Vinehout, Michael W. Dailey

**Affiliations:** 1 New York State Department of Health, AIDS Institute, New York, USA; 2 New York State Division of Criminal Justice Services, Albany, USA; 3 Department of Emergency Medicine, Albany Medical College, Albany, USA

**Keywords:** Naloxone, Narcan, Police, Law enforcement, EMS, Prehospital, Harm reduction, Opioid overdose, New York

## Abstract

**Background:**

The COVID-19 pandemic has amplified the need for wide deployment of effective harm reduction strategies in preventing opioid overdose mortality. Placing naloxone in the hands of key responders, including law enforcement officers who are often first on the scene of a suspected overdose, is one such strategy. New York State (NYS) was one of the first states to implement a statewide law enforcement naloxone administration program. This article provides an overview of the law enforcement administration of naloxone in NYS between 2015 and 2020 and highlights key characteristics of over 9000 opioid overdose reversal events.

**Methods:**

Data in naloxone usage report forms completed by police officers were compiled and analyzed. Data included 9133 naloxone administration reports by 5835 unique officers located in 60 counties across NYS. Descriptive statistics were used to examine attributes of the aided individuals, including differences between fatal and non-fatal incidents. Additional descriptive analyses were conducted for incidents in which law enforcement officers arrived first at the scene of suspected overdose. Comparisons were made to examine year-over-year trends in administration as naloxone formulations were changed. Quantitative analysis was supplemented by content analysis of officers’ notes (*n* = 2192).

**Results:**

In 85.9% of cases, law enforcement officers arrived at the scene of a suspected overdose prior to emergency medical services (EMS) personnel. These officers assessed the likelihood of an opioid overdose having occurred based on the aided person’s breathing status and other information obtained on the scene. They administered an average of 2 doses of naloxone to aided individuals. In 36.8% of cases, they reported additional administration of naloxone by other responders including EMS, fire departments, and laypersons. Data indicated the aided survived the suspected overdose in 87.4% of cases.

**Conclusions:**

With appropriate training, law enforcement personnel were able to recognize opioid overdoses and prevent fatalities by administering naloxone and carrying out time-sensitive medical interventions. These officers provided life-saving services to aided individuals alongside other responders including EMS, fire departments, and bystanders. Further expansion of law enforcement naloxone administration nationally and internationally could help decrease opioid overdose mortality.

## Background

### The opioid crisis in the New York State

In the past decade, there have been dramatic increases in drug-related deaths in the United States (US). Drug overdose is a leading cause of injury-related death in the US, accounting for more than 750,000 deaths from 1999 to 2018, 60% of which involved an opioid [1–3]. While there was a slight decrease in opioid overdose mortality in the US from 2017 to 2018 [1, 4], the number of overdose deaths once again increased in 2019, specifically driven by illicitly manufactured fentanyl and its analogs [5]. Moreover, provisional data suggest that overdose mortality in the US has been exacerbated by the coronavirus disease 2019 (COVID-19) pandemic [6–11]. More than 81,000 drug overdose deaths occurred in the US in the 12 months ending in May 2020, breaking the record for the highest number of overdose deaths within a 12-month period [12].

In NYS, the age-adjusted rate of deaths involving all opioids increased over threefold from 4.9 to 16.5 per 100,000 between 2010 and 2017 and remained elevated at 14.9 per 100,000 through 2019. New York State Department of Health (NYSDOH) quarterly county-level reports indicate the upsurge of opioid overdose since the emergence of the COVID-19 pandemic [13]. From September 2020 through September 2021, over 3000 lives were lost due to opioid overdose in NYS, over a 7% increase from the previous 12-month period, and a 36.7% increase since the period ending in September 2019, prior to the COVID-19 pandemic [14].

The need for overdose prevention and intervention continues to grow, particularly given the social and psychological impacts of the COVID-19 pandemic [6, 15–17]. The life-threatening characteristic of an opioid overdose is a decline in respiratory function which may lead to organ damage including brain death and cardiac arrest. Naloxone is a fast-acting, effective antidote for opioid overdose that safely reverses opioid poisoning and restores normal breathing [18]. Naloxone can cause opioid withdrawal in dependent individuals; generally, it is limited to short-term discomfort but may be associated with pulmonary complications and other unintended consequences, particularly at higher doses [18, 19]. The United States Food and Drug Administration (FDA) first approved naloxone in 1971, and it has been routinely used in ambulances and emergency departments to treat opioid-related overdose since then.

### New York State efforts to reduce opioid overdose mortality

Most opioid-related overdoses are reversible if witnessed and responded to promptly. Many overdoses occur with at least one other person present and the timely administration of naloxone by bystanders can save lives [20]. In 2006, New York Public Health Law §3309 and associated regulations permitted eligible organizations registered with the NYSDOH to train potential witnesses to an opioid overdose on identifying and responding to such incidents with the administration of naloxone [21]. In the same year, NYSDOH launched the Opioid Overdose Prevention Program (OOPP), an initiative to train community members through registered programs in recognizing overdoses and in responding to them by administering naloxone. This program works to increase naloxone access “for ALL community members—regardless of [their] level of understanding/knowledge about opioid overdose” [22].OOPP allows all trained NYS community persons, including opioid-dependent individuals and their family and friends, to receive two doses of free naloxone after completing a brief training.

As the opioid crisis worsened, New York State’s response expanded to include registered programs for emergency medical technicians and paramedics, law enforcement personnel, and firefighters building on community naloxone provision as well as decades of naloxone administration by EMS. In 2020, NYSDOH received 19,806 records of naloxone administration in the community among which 80.0% was administered by EMS, 10.2% by laypersons, and 8.9% by law enforcement [23]. As of July 2022, NYSDOH has received records of 16567 instances of naloxone administration by laypersons, 11,683 by law enforcement, and 1385 by the fire department personnel since the inception of these programs. Unlike public safety agencies, community members are not mandated to report reversals and there is no uniform mechanism for reporting, other than through registered programs. Syringe access programs reported 68% of reversals were by laypersons.

### Law enforcement naloxone administration program

In 2014, a change in the law allowed naloxone to be provided to individuals under “standing orders” (non-patient-specific prescriptions). This expansion to the law meant both lay persons and pharmacists could train and offer naloxone without an on-site prescriber. These legislative changes also allowed distributed naloxone to be shared, so that naloxone could be placed in police cars with other first aid equipment, rather than assigned to individual officers, similar to the availability of automated external defibrillators carried by some law enforcement [24].

The law enforcement program of naloxone administration follows the same procedures as OOPP. Naloxone administration is voluntary and law enforcement agencies do not receive any additional support for this activity, other than NYSDOH-purchased naloxone. To address possible hesitation in reporting observed overdose cases to 911, New York State’s Good Samaritan Law (GSL) went into effect in 2011. This law shields both the overdosed person and those summoning aid from charge and prosecution for possession of most controlled substances [21]. Similar to the OOPP community programs, police agencies must register with NYSDOH or the New York City Department of Health and Mental Hygiene (NYCDOHMH) to receive free naloxone for their trained staff. Naloxone can be administered by injection or intranasally. However, nasal administration is preferred by law enforcement and many community programs as it can be easily administered by laypersons and does not involve the use of, or exposure to, needles.

NYS started its statewide law enforcement naloxone initiative in 2014 through a partnership among state agencies overseeing health, drug treatment, and criminal justice services, as well as with a non-profit harm reduction agency and a medical college. Elements of this initiative included developing and delivering training, supplying naloxone, providing implementation guidance, and having a system for collecting data on naloxone administrations.

**Fig. 1:**
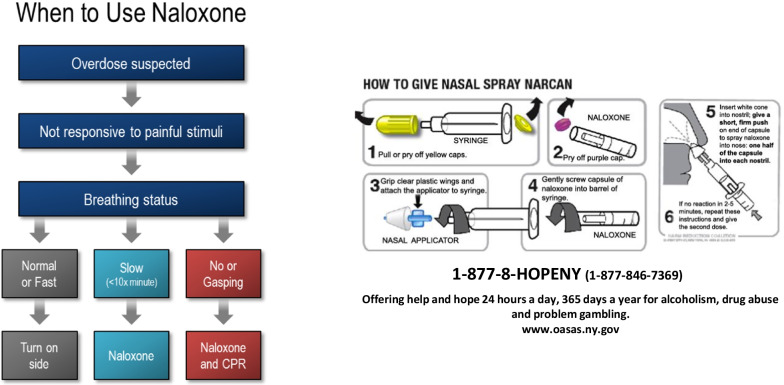
Naloxone administration pocket card for law enforcement personnel

Between October 2014 and July 2020, 12,400 law enforcement personnel were trained through the initiative by NYSDOH [25]. The training included information on the State’s syringe access programs, medications for opioid use disorder, the legality of syringe and naloxone possession, harm reduction approaches to substance use, the 911/GSL, the signs of an opioid overdose, how to administer intranasal naloxone, and assess whether an additional dose of naloxone is necessary. A simple pocket card was developed to support the training (see Fig. 1).

Despite the rapid expansion of the law enforcement administration of naloxone across the country, there are few systematic reviews of those efforts covering a large geographic area in peer-reviewed literature. Other studies have validated that trained law enforcement personnel can identify opioid overdoses and effectively administer intranasal naloxone [26–31]. The data in those studies were collected and analyzed for relatively short periods of time [27, 29, 31]. This study examines 6 years of data on law enforcement naloxone administrations in a large state with significant socio-demographic diversity and a wide range of population density.

## Methods

Officers complete a usage report form after each administration of naloxone in the field and submit the form to NYSDOH for data entry, analysis, and reporting. To examine circumstances and outcomes surrounding law enforcement-administered naloxone in NYS, we analyzed all law enforcement-reported instances of suspected opioid overdose in which NYS law enforcement officers administered at least one dose of naloxone between January 1, 2015, and December 31, 2020. The naloxone usage report forms submitted by officers collect information on the agency with which the officer is affiliated, the date, time, county, and ZIP code of the suspected opioid overdose, the age and perceived gender of the aided individual, the time between the arrival of law enforcement and EMS, the observed signs and symptoms before and after the administration of naloxone, whether the aided was transferred to a hospital, substances believed to have been used, the number of naloxone doses administered by the responding officer as well as those administered by other public safety personnel and bystanders, and whether the aided survived the suspected overdose. Substances reported were based upon police observation in the field, disclosure by the aided individuals or bystanders, and symptoms of the aided. Toxicology tests were not conducted at the scene. Information on survival status is not always available if the aided individual was transported by EMS with ongoing intervention. The report form also includes an open-ended field for additional notes, which officers usually use for their observation and summary of the event.

This analysis includes instances where police participated in the overdose response by administering at least one dose of naloxone. Naloxone administration reports missing incident date and location were excluded from analysis. Instances where an incident was reported more than once were deduplicated through case-by-case examination of the reports. When an opioid overdose was reported by more than one agency involved in the incident, duplicates were determined based on the time and location (i.e., ZIP code) of the overdose and the characteristics of the aided individual.

Descriptive statistics were calculated to characterize suspected overdose instances in which law enforcement-administered naloxone including mean and standard deviation of the number of naloxone doses administered, reported age and sex of aided individuals, substances used by aided individuals, and time of arrival of law enforcement and EMS. Additional information reviewed for all cases included administration of naloxone by others (e.g., laypersons, EMS), additional response actions taken by officers at the scene, whether care was transferred to EMS, and whether the aided survived. The instances in which law enforcement officers arrived prior to any other public safety personnel were reviewed to determine the mean and standard deviation of time between law enforcement and EMS arrival along with whether care was transferred to EMS.

Appropriateness of naloxone administration in all cases and in cases where law enforcement officers arrived first was assessed by reviewing characteristics of the aided individual prior to naloxone administration including responsiveness and breathing. In instances that may have indicated inappropriate naloxone administration, officers’ notes were reviewed to provide additional context. Suspected overdose incidents were then reviewed separately for non-fatal and fatal incidents, and characteristics of the aided prior to naloxone administration were described. Throughout the quantitative analysis, the contents of the officers’ notes about the incidents were used as supplemental qualitative information to provide more context to the suspected overdose incidents.

## Results

**Fig. 2:**
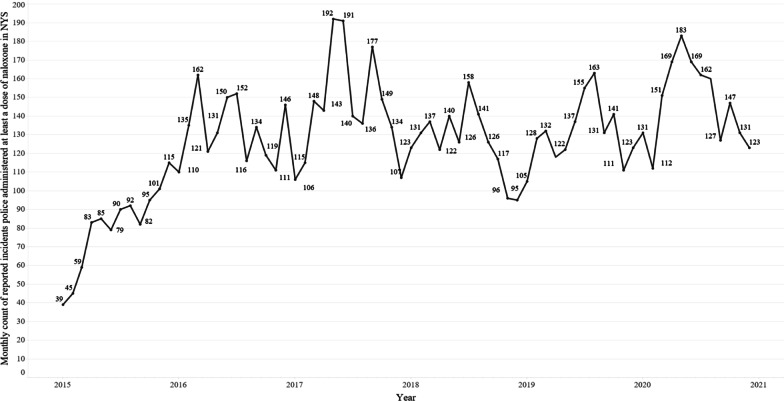
Naloxone administration by law enforcement in NYS (2015–2020)

After the necessary exclusions and deduplication, the analytical sample includes 9133 forms completed by 5835 unique officers from 434 state, county, municipal, and public authority law enforcement agencies located in 60 counties across NYS [25]. There were 2192 supplemental comments made by officers completing the forms. Consistent with opioid overdose trends in NYS, naloxone administration by law enforcement peaked in 2017 and slightly declined in 2018 and 2019. Administration of naloxone by law enforcement peaked again by mid-2020, showing a 13.1% increase of naloxone usage reports compared to 2019 (Fig. 2).

Police officers reported the administration of an average of 2 naloxone doses to the aided (standard deviation (SD) = 1). The mean age of the aided in this study was 34.3 years (SD = 11.8); 70.2% of the aided were perceived as male and 29.3% as female by the officers who attended the overdose scene (Table 1). Heroin was suspected in 64.2% of the reported cases, though not necessarily as the only substance. Only 2.3% (*n* = 212) of the reported overdoses were believed to involve the consumption of medication for opioid use disorder (MOUD) such as methadone (*n* = 92) or buprenorphine (*n* = 120). Among these few cases that reported MOUD use, 52.2% involved other suspected substances, including heroin (35.1%), benzodiazepines (11.7%), and cocaine (5.4%). Substances reported were based upon police observation in the field, disclosure by the aided or bystanders, or symptoms of the aided. Toxicology tests were not conducted.

**Table 1:** Pre-naloxone administration symptoms and survival among all aided individuals

	Total	Slow breathing or not breathing at all	Normal breathing	Fast breathing	Missing breathing status	Unresponsive	Responsive but sedated	Alert	Missing responsiveness status	Mean of total number of naloxone doses
*All cases of police naloxone administration*										
Number	9133	8652	195	150	136	8628	358	32	115	1.95
Percent	100.0	94.7	2.1	1.6	1.5	94.5	3.9	0.4	1.3	n/a

	Number (percent)	Number (percent)	Number (percent)	Number (percent)	Number (percent)	Number (percent)	Number (percent)	Number (percent)	Number (percent)	Number
Survived	7981 (87.4)	7551 (87.3)	184 (94.4)	132 (88.0)	114 (83.8)	7522 (87.2)	326 (91.1)	31 (96.9)	102 (88.7)	1.94
Did not survive	620 (6.8)	607 (7.0)	1 (0.5)	1 (0.7)	11 (8.1)	608 (7.0)	8 (2.2)	0 (0)	4 (3.5)	1.98
Not known	532 (5.8)	494 (5.7)	10 (5.1)	17 (11.3)	11 (8.1)	498 (5.8)	24 (6.7)	1 (3.1)	9 (7.8)	2.00
All cases of police naloxone administration when police arrived first										
Number	7845	7431	156	142	116	7396	339	29	81	1.96
Percent	100.0	94.7	2.0	1.8	1.5	94.3	4.3	0.4	0.1	n/a
Survived	6870 (87.6)	6519 (87.7)	150 (96.2)	126 (88.7)	75 (64.7)	6481 (87.6)	298 (87.9)	28 (96.6)	63 (77.8)	1.90
Did not survive	520 (6.6)	509 (6.8)	1 (0.6)	0 (0)	10 (8.6)	510 (6.9)	6 (1.8)	0 (0)	4 (4.9)	1.97
Not known	455 (5.8)	403 (5.4)	5 (3.2)	16 (11.3)	31 (26.7)	405 (5.5)	35 (10.3)	1(3.4)	14 (17.3)	2.0

The initiative being studied is specially intended for situations where law enforcement personnel arrive at the scene before other public responders and therefore can provide lifesaving medication for opioid poisoning. Police arrived first on the scene in 85.9% of the cases reported by law enforcement in which they administered naloxone (*n* = 7845). In some instances, police encountered the incident while on patrol: “Aided was found unconscious on [a] platform at 125th Street and Lenox Train Station due to an overdose of an unknown substance.” When police arrived first, the average time until EMS arrival was 5.95 min^1^ (median: 5.0 min). In these instances, officers took note of the aided’s symptoms and examined the scene. If they determined there was a probability that the incident involved an opioid overdose, they administered naloxone and performed other life-saving actions before care was transferred to EMS.

Consistent with their training, officers assessed the possibility of an opioid overdose based on the aided individual’s breathing and responsiveness, as well as information from others and drug-related observations from the scene, such as the presence of paraphernalia. In 64.0% of cases where police arrived first on the scene, the aided individual was described as *breathing slow[ly]* (*n* = 5024) and another 30.6% as *appearing not to breathe* (*n* = 2407). In nearly 95% of incidents where police arrived first at the scene (*n* = 7396) the aided individual was unresponsive, and 4.3% (*n* = 339) were responsive but sedated when the officer arrived. When police arrived first on the scene, the aided was transported to a hospital 88.9% of the time (*n* = 6974).

We examined pre-naloxone-aided symptoms to see if the police administered naloxone according to their training. In 94.7% the aided was described as breathing slowly, or as seeming to not breathe at all. In the remaining 5.3% (*n* = 482) incidents, breathing was reported as normal (2.1%, *n* = 195), fast (1.6%, *n* = 150), or the form did not include breathing status (1.5%, *n* = 136). Taking a closer look at these incidents, we looked at other characteristics such as aided responsiveness. Moreover, we checked whether the police arrived before EMS on the scene, indicating that they were key decision-makers about naloxone administration in those circumstances.

We found that when police attended the incident before EMS, in only 0.1% (*n* = 10) of cases the aided did not show symptoms of breathing suppression nor was unresponsive, contradicting with typical physiological symptoms of a person who is experiencing overdose. We further investigated each of these reports to gain more contextual information from the incidents. In one of these instances, the additional note indicated that “[the] aided injected narcotics into his neck attempting to commit suicide… [the] aided was administered 8[mg] of Narcan by [name of the police officer] and transported to [hospital].” In another case, the aided individual’s wife had notified the attending police officer of the opioid use, and she had already administered one dose prior to police arrival. The remaining eight cases were not accompanied by additional notes.

The excerpts below are from the officers’ notes when they arrived first on the scene. The notes provide context to their assessment of the incidents. The selected notes represent the majority of naloxone administration cases where the aided demonstrated had slow breathing, appeared not to be breathing at all, or was unresponsive.

- “Aided [was] found unconscious, blue in face, with faint pulse, belt around left arm, and syringe on dresser.”
- “Aided found supine in store, unconscious, unresponsive, snoring, pinpoint pupils. Staff reports aided collapsed after buying pain med prescription. Track marks noted on both arms…”.

In 37% of cases (*n* = 3359) the reporting law enforcement officer observed the aided receiving at least one additional dose of naloxone by another party including a layperson (in many cases a family member or friend of the aided), a firefighter, or EMS personnel. The officers took note of the naloxone dosage the aided individual had reportedly received prior to their arrival and administered additional naloxone if needed. Based on their training and available equipment, law enforcement personnel also performed other lifesaving actions in addition to naloxone administration including, but not limited to, performing a sternal rub in nearly 60% of cases, using a bag valve mask in nearly 20% of cases, chest compressions in nearly 14% of cases, and deploying a defibrillator in nearly 8% of the reported cases.

The following officer notes showcase examples where the police worked with other parties to provide post-overdose care for the aided:

- “[I]nitial two doses of Narcan [were] administered by bystander, CPR was performed by [a] bystander, while I [police officer] attached [automated external defibrillator], I administered my first (patient’s third) dose of Narcan which restored [their] breathing; after several minutes [the] patient became responsive.”
- “Upon arrival, [a] bystander [was] doing CPR, assessment revealed the male had a pulse with labored breathing. Naloxone was administered [by law enforcement] just prior to EMS arrival; 1 dose given by [the] bystander, 1 dose given by officer, 2 given by EMS.”
- “[The] Aided [was] found lying on his back, unresponsive, with slow, distressed breathing and discolored skin. [Law enforcement] officer administered naloxone and EMS administered oxygen.”

The reporting law enforcement personnel indicated that 87.4% of the time (*n* = 7981) the aided survived the suspected overdose, 6.9% (*n* = 629) were deceased, and the survival status was unknown for 5.7% (*n* = 523). Among the cases where survival was reported (*n* = 7981), 86.0% of the time the police arrived first on the scene (*n* = 6870). Among those who survived and had disposition information (*n* = 7876), in 91.2% of instances law enforcement transferred care to EMS (*n* = 7187), and in 8.3% of instances the aided refused to be transported to the hospital by EMS (*n* = 654). In the remaining 0.3% of cases (*n* = 22), the aided was transported by the police.

In approximately three percent (*n* = 15) of the cases who were deceased when police arrived first, the aided was determined and noted to be dead upon arrival of the police officer. For example, one officer wrote, “Naloxone administered before checking vitals based on drug paraphernalia found on scene. Aided had overdosed a few hours prior, was dead already.” In 89.0% (*n* = 463) of the deceased cases where police arrived first, the aided was described as neither responsive nor breathing when the officer arrived on the scene. Among these, 56% (*n* = 261) were pronounced dead at the hospital, and in 44% (*n* = 202) of the cases, the aided was not transferred to the hospital, most likely because they were pronounced dead on the incident scene by EMS either by protocol or through medical consultation: “Before officer could administer a second dose, EMS arrived and declared aided deceased.”

Among the cases who did not survive the suspected opioid overdose, only 5% (*n* = 27) appear to have been alive when the police arrived before the EMS (*n* = 520). This is determined through the indication that either the aided was observed to be still breathing, or they appeared as being responsive but sedated when the officer entered the scene. Among these 27 cases, 6 were marked as responsive but sedated and the remaining 21 as unresponsive upon the arrival of the officer to the scene. Evidence of heroin, crack cocaine, and opioid analgesics were observed on the scene in 33%, 11%, and 11% of the cases, respectively. The average dose of law enforcement-administered naloxone for this group was 2 vials. In 15% of the cases (*n* = 4) at least one additional dose of naloxone was administrated by EMS, in 7.5% (*n* = 2) by a bystander, and in 4% (*n* = 1) by fire personnel.

Naloxone administration reports were mostly conducted at or shortly after the incident. Therefore, the cause of death is not confirmed in these reports; they are reported by police, EMS, or emergency department physicians based upon the initial examination of the aided’s symptoms or the scene. As can be drawn from the additional notes by the administering officers, police sometimes used naloxone as a cautionary action even when they did not identify opioid use on the scene. For example, one officer noted that “Subject [was] found unresponsive with no pulse and not breathing. Narcan [was used] as a precaution in the event the subject [had an opioid] overdose.”

It is likely that not all reported fatalities were due to opioid overdose, even when opioid use was observed on the scene. For example, one officer noted: “[a]ided was the victim of multiple stab wounds. Information was relayed to patrol that victim had possibly also used heroin and crack. Victim succumbed to his injuries” or in another case the aided had “blood on [a] white t-shirt and right arm.” Another case is noted as a “possible heart attack” and through another description, the “attending physician believe[d] that the person overdosed on a non-opioid substance. [therefore, the] naloxone delivered by reporting officer and by the doctor at the hospital was ineffective.”

## Discussion

Whether police should be involved in naloxone administration is debated in the literature. The first program of naloxone administration by law enforcement in the US started in 2010 in Massachusetts, and by March 2018, 2300 law enforcement agencies in 42 states were administering naloxone [32], representing approximately 12% of police agencies across the US [33].

Amid the expansion of this initiative, critics point to barriers of engaging law enforcement in overdose response and call for limiting police engagement in substance use programs. Some scholars suggest that police involvement in overdose treatment introduces new risks of harm including further criminalization of substance use [34]. The presence of police may hinder emergency reporting of overdose due to the public mistrust of police rooted in the historical coercion of the criminal justice system in the lives of people who use drugs, particularly among Black and Hispanic communities [35, 36]. Studies show that despite the enactment of GSLs in most states, many bystanders are reluctant to call 911 on the scene of an overdose out of fear that police will arrive and arrest them or the person who overdosed for possession of drugs [35, 37–39]. Moreover, the administration of naloxone by laypeople is the most cost-effective method to save lives [40].

On the other hand, as shown in our program, there is evidence that naloxone administration by police can be a practical strategy to reduce overdose mortality [26, 41–45]. In many localities, especially in rural settings, law enforcement officers are often the first to respond to 911 calls for suspected opioid overdose [27, 46]. The capacity of law enforcement personnel to respond quickly to an opioid overdose often exceeds that of other first responders [32]. In addition, law enforcement officers on patrol may encounter overdoses which were not already reported through the emergency dispatch system.

This review of the NYS law enforcement naloxone initiative uses multi-year data that cover a large geographic region including urban, suburban, and rural areas. Our findings suggest that police can effectively assess the signs of a suspected opioid overdose and administer life-saving naloxone appropriately and in accordance with the training they received [46, 47]. Nearly 95% of the naloxone recipients by police showed respiratory depression, the most important factor that identifies an opioid overdose and the main cause of death due to opioid overdose.

Our results align with a recent study from Tempe, Arizona, where police video footage of body-worn cameras was studied to examine what transpired in 168 cases when police attended an overdose scene. The Tempe study shows that when appropriate officer trainings and legal protections for the aided and bystanders are available, police administration of naloxone can be an effective strategy to respond to opioid mortality [44].

The question remains whether the presence of police at overdose scenes may hinder overdose reporting by bystanders. The literature on the role of GSL in 911 reporting of overdose is mixed and context-specific [48]. The barrier to overdose reporting can be partly explained by lack of awareness of GSL among people who use drugs [49]. In New York, the odds of bystander calling 911 at an overdose scene was tripled when participants had correct knowledge of GSL [50]. However, other studies from Baltimore, MD show despite awareness of GSL, people who use drugs (PWUD) continue to fear police involvement in overdose scenes—especially if they are Hispanic or Black [35, 51, 52].

The two camps offer different solutions to overcome 911 reporting barriers; one group emphasizes on the need to increase awareness about GSL for PWUD and law enforcement personnel; the other group argues that police non-response in overdose incidents would bring about better health outcomes for people who use drugs unless a specific security risk is present at the scene. Instead of law enforcement, the latter group call for the engagement of social workers and community members in naloxone administration efforts [39, 51].

Naloxone is a safe medication, and the expansion of naloxone administration training and use among all groups of community members helps to decrease mortality for people using opioids. NYSDOH has taken a multipronged approach to increase the probability of naloxone usage at overdose scenes by utilizing the existing infrastructure including law enforcement. To respond to the social marginalization of PWUD, NYSDOH’s strategy has been to partner with police, provide continued training on naloxone administration, and conduct regular program monitoring and evaluation of partnership programs. As an example, the data used in this study have been collected and regularly analyzed for internal quality control of the law enforcement administration of naloxone in New York State.

Moreover, the New York State implementation of naloxone administration by law enforcement does not disrupt nor replace the existing community-based and EMS programs. On the contrary, the law enforcement program was implemented as a specialized program among others under the Opioid Overdose Prevention Program (OOPP). In NYS, law enforcement agencies do not receive any incentives or additional funding for administering naloxone, and there is little or no evidence that police involvement reduces the engagement of other groups in overdose reversal efforts. Similar to community organizations, police agencies must register with NYSDOH or NYCDOHMH to receive naloxone.

The use of naloxone by the police is most helpful in instances where the aided is directly discovered by the police on patrol, or when the police arrive first to the scene, which was the case in the large majority of reported incidents [53, 54]. Law enforcement naloxone programs are of benefit throughout NYS, as rapid intervention in opioid overdose is key to optimal outcomes and EMS responses may be delayed anywhere, including rural environments, broadly dispersed suburban neighborhoods, and urban traffic patterns and vertical patrol areas, such as high-rise structures and subways.

As part of this initiative, more than 12,000 law enforcement personnel learned about syringe access and medication for opioid use disorder. These officers also received a focused review of the 911/GSL. The data present successful instances where the police provided care to individuals who had overdosed. This care included naloxone administration, EMS notification, transportation to the hospital, and conducting basic first aid services such as cardiopulmonary resuscitation (CPR). It is hoped that successful encounters can help repair the historically difficult relationship between PWUD and police and decrease bystander hesitation to call 911 [55]. Those successful encounters may also improve job satisfaction among police and improve community relations between police and the lay public [28, 32, 56, 57].

The number of doses of naloxone that aided individuals received remained stable throughout the study. This is noteworthy because midway through the study period the deployed formulation changed resulting in a doubling of each dose from 2 mg (2 mg/2 ml) to 4 mg (4 mg/0.1 ml) yielding a much higher effective administration of medication [58]. The 2 mg formulation also entailed multi-step assembly to convert a naloxone vial intended for parenteral administration into one that could be used for nasal administration using a separate atomizer. The 4 mg product requires no assembly and is much easier to deploy rapidly. The times recorded on the administration forms are retrospective, and by anecdotal report, the standard process for many officers was for the second dose to be administered directly after the first, often not allowing time for the first to take effect. Therefore, because the 2 mg formulation had a longer time interval between arrival and nasal administration of less concentrated formula, both the administration and the absorption of the medication would take longer and potentially would be erratically absorbed [59]. Because of the decreased time to administration of the second dose using the 4 mg no-assembly formulation and its more rapid deployment, we posit that there is no change in the amount of naloxone required for the reversal of the overdoses.

Due to the nature of the program and the reporting process, several limitations to the data and analysis should be noted. An important limitation to the data and analysis is the absence of analysis on the racial composition of the aided [51]. Information on racial composition was not collected since it is customary for the aided to be found unresponsive on the scene and reporting must be based on officers’ perceptions, hence unreliable. Future research could review the racial patterns in the administration of naloxone by police.

Second, we did not have access to arrest data to ascertain whether any of the aided or bystanders were arrested after overdose prevention and reversal activity was completed. Future research should explore how the GS are practiced in the overdose scenes. Third, we did not have data on the number of overdose incidents that were not reported to 911, neither are we aware of the number of police officers who attended the overdose scene yet may have hesitated to use naloxone, despite the presence of symptoms in the aided. Fourth, although naloxone administration reporting is strongly encouraged as a matter of policy, it is not universally practiced among officers in the field, and reports may not always be submitted in a timely manner. Additionally, New York City is largely underrepresented in our data as agencies in New York City are registered with NYCDOHMH which receives their naloxone reports. Fifth, data are self-reported by the responding officer, resulting in the possibility of errors in reporting or missing data, as it is collected at the scene of a suspected overdose during a medical emergency. Of note, follow-up inquiries were made by NYSDOH to participating law enforcement agencies when core information was missing or deemed contradictory on the reporting forms. Concerted efforts have been made to ensure highest data quality available for program review and improvement. A new online reporting system is currently under development and going through pilot testing to minimize data errors and improve timeliness of reporting.

Sixth, there was a change in the formulation of the naloxone distributed to law enforcement from a multi-stage 2 mg administration to a 4 mg single-stage device in 2016–2017, with updated training, which may contribute to a change in implementation. However, this formulation change is not reflected in the data used for this study. Future research should examine the naloxone dosage received by the aided and the police timing in between the doses. Lastly, the cases presented in this study do not include toxicology testing or hospital discharge information; therefore, it is possible that naloxone is administered in instances other than opioid overdose.

## Conclusions

The role of law enforcement in responding to overdoses in communities continues to be debated. One side of the debate opposes the involvement of law enforcement in naloxone administration, considering the war on drugs historical context. On the other hand, the proponents of engaging police in overdose reversal activities refer to the pragmatic benefits, such as the ability to use an already established infrastructure to save lives. New York State’s first intervention to address drug user health started in 1992 with the launch of the syringe exchange program. Over the past 15 years, NYS has developed and implemented naloxone programs in response to the opioid crisis, including programs designed for community members (since 2006), basic emergency medical technicians (since 2012), law enforcement (since 2014), non-EMS firefighters (since 2015), and most recently, “leave-behind” programs for distribution of naloxone following community encounters with people who use drugs by EMS providers and sheriff’s deputies [47, 56]. While complementing one another, each of these programs plays a distinct role in responding to suspected overdose cases, and law enforcement naloxone administration does not deny nor substitute the need for lay persons’ access to the lifesaving treatment, rather, equipping law enforcement with naloxone serves as a safety net. This study presents evidence that police can successfully recognize symptoms of a possible opioid overdose, administer naloxone, and save lives. We recommend future research to examine how the experience of overdose reversal may influence the relationship dynamics between PWUD and law enforcement and whether their mutual experience helps repair or widen the fissure due to the criminalization of substance use.

## Data Availability

The dataset used and analyzed during the current study are available from the corresponding author on reasonable request.

## Abbreviations

COVID-19: Coronavirus disease 2019
CPR: Cardiopulmonary resuscitation
EMS: Emergency medical services
FDA: United States Food and Drug Administration
GSL: Good Samaritan Law
MOUD: Medication for opioid use disorder
NYCDOHMH: New York City Department of Health and Mental Hygiene
NYS: New York State
NYSDOH: New York State Department of Health
OOPP: Opioid Overdose Prevention Program
PWUD: People who use drugs
SD: Standard deviation
US: United States

## Footnotes

1 To calculate the mean for the time difference between the emergency medical services and police on the scene, the outlier incidents where EMS was reported to be arriving three standard deviations from the mean were removed from the data.

## References

[1] Centers for Disease Control and Prevention (CDC), National center for health statistics. Wide-ranging online data for epidemiologic research (WONDER) 2020. http://wonder.cdc.gov.

[2] CDC. The drug overdose epidemic: behind the numbers 2020. https://www.cdc.gov/opioids/data/index.html.

[3] CDC. Opioids data analysis & resources 2021. https://www.cdc.gov/opioids/data/analysis-resources.html.

[4] Wilson NKM, Seth P, Smith H, Davis NL (2020). Drug and opioid-involved overdose deaths: United States, 2017–2018. MMWR Morb Mortal Wkly Rep.

[5] O'Donnell J, Gladden RM, Mattson CL, Hunter CT, Davis NL (2020). Vital signs: characteristics of drug overdose deaths involving opioids and stimulants—24 states and the district of Columbia, January–June 2019. MMWR Morb Mortal Wkly Rep.

[6] Henry BF, Mandavia AD, Paschen-Wolff MM, Hunt T, Humensky JL, Wu E (2020). COVID-19, mental health, and opioid use disorder: old and new public health crises intertwine. Psychol Trauma.

[7] Volkow ND (2020). Collision of the COVID-19 and addiction epidemics. Ann Intern Med.

[8] Becker WC, Fiellin DA (2020). When epidemics collide: coronavirus disease 2019 (COVID-19) and the opioid crisis. Ann Intern Med.

[9] Alexander GC, Stoller KB, Haffajee RL, Saloner B (2020). An epidemic in the midst of a pandemic: opioid use disorder and COVID-19. Ann Intern Med.

[10] Stephenson J (2020). Drug overdose deaths head toward record number in 2020, CDC warns. JAMA Health Forum.

[11] American Medical Association Advocacy Resource Center. Issue brief: Reports of increases in opioid-related overdose and other concerns during COVID pandemic. 2020.

[12] Overdose Deaths Accelerating During COVID-19 [press release]. www.cdc.gov. 2020.

[13] Scholl LSP, Kariisa M, Wilson N, Baldwin G (2019). Drug and opioid-involved overdose deaths: United States, 2013–2017. MMWR Morb Mortal Wkly Rep..

[14] New York State Department of Health (NYSDOH). New York State: county opioid quarterly report published July, 2020. https://www.health.ny.gov/statistics/opioid/data/pdf/nys_jul20.pdf.

[15] Chiappini S, Guirguis A, John A, Corkery JM, Schifano F (2020). COVID-19: the hidden impact on mental health and drug addiction. Front Psychiatry.

[16] Linas BP, Savinkina A, Barbosa C, Mueller PP, Cerdá M, Keyes K (2021). A clash of epidemics: impact of the COVID-19 pandemic response on opioid overdose. J Subst Abuse Treat.

[17] Holland KM, Jones C, Vivolo-Kantor AM, Idaikkadar N, Zwald M, Hoots B (2021). Trends in US emergency department visits for mental health, overdose, and violence outcomes before and during the COVID-19 pandemic. JAMA Psychiat.

[18] Boyer EW (2012). Management of opioid analgesic overdose. N Engl J Med.

[19] Dale O (2022). Pharmacokinetic considerations for community-based dosing of nasal naloxone in opioid overdose in adults. Expert Opin Drug Metab Toxicol.

[20] Bohnert AS, Tracy M, Galea S (2012). Characteristics of drug users who witness many overdoses: implications for overdose prevention. Drug Alcohol Depend.

[21] NYSDOH. Opioid overdose prevention programs, Section 80.138 regulations 2021. https://www.health.ny.gov/diseases/aids/general/opioid_overdose_prevention/regulations.htm.

[22] NYSDOH. Community calendar of opioid overdose trainings 2022. https://www.health.ny.gov/diseases/aids/general/opioid_overdose_prevention/training_calendar.htm.

[23] NYSDOH. New York State opioid annual report 2021. 2021. https://www.health.ny.gov/statistics/opioid/data/pdf/nys_opioid_annual_report_2021.pdf

[24] NYSDOH. New York State’s opioid overdose regulations questions and answers 2015. https://www.health.ny.gov/diseases/aids/general/opioid_overdose_prevention/questions_answers.htm.

[25] NYSDOH. The New York State opioid overdose and intranasal naloxone program for law enforcement 2021; (Data Brief #5). https://www.health.ny.gov/diseases/aids/general/opioid_overdose_prevention/docs/fact_sheet_law_enforcement.pdf.

[26] Berardi L, Bucerius S, Haggerty KD, Krahn H (2021). Narcan and Narcan't: Implementation factors influencing police officer use of Narcan. Soc Sci Med.

[27] Fisher R, O'Donnell D, Ray B, Rusyniak D (2016). Police officers can safely and effectively administer intranasal naloxone. Prehosp Emerg Care.

[28] Purviance D, Ray B, Tracy A, Southard E (2017). Law enforcement attitudes towards naloxone following opioid overdose training. Subst Abus.

[29] Kitch BB, Portela RC (2016). Effective use of naloxone by law enforcement in response to multiple opioid overdoses. Prehosp Emerg Care.

[30] Lowder EM, Lawson SG, O'Donnell D, Sightes E, Ray BR (2020). Two-year outcomes following naloxone administration by police officers or emergency medical services personnel. Criminol Public Policy.

[31] Saratoga County EMS Council. Leave behind naloxone program. 2020. https://saratogaems.org/wp-content/uploads/2020/02/LeaveBehindNaloxone-SaraCounty2020.pdf.

[32] Lurigio AJ, Andrus J, Scott CK (2018). The opioid epidemic and the role of law enforcement officers in saving lives. Vict Offender.

[33] Banks D, Hendrix J, Hickman M, Kyckelhahn T (2016). National sources for law enforcement employment data.

[34] Doe-Simkins M, El-Sabawi T, Carroll JJ (2022). Whose concerns? It’s time to adjust the lens of research on police-involved overdose response. Am J Public Health.

[35] Lopez AM, Thomann M, Dhatt Z, Ferrera J, Al-Nassir M, Ambrose M, Sullivan S (2022). Understanding racial inequities in the implementation of harm reduction initiatives. Am J Public Health.

[36] Latimore AD, Bergstein RS (2017). “Caught with a body” yet protected by law? Calling 911 for opioid overdose in the context of the Good Samaritan Law. Int J Drug Policy.

[37] Nguyen H, Parker BR (2018). Assessing the effectiveness of New York’s 911 good Samaritan law-evidence from a natural experiment. Int J Drug Policy.

[38] Follett KM, Piscitelli A, Parkinson M, Munger F. Barriers to calling 9-1-1 during overdose emergencies in a Canadian context. Crit Soc Work. 2014;15(1).

[39] van der Meulen E, Chu SK, Butler-McPhee J (2021). That’s why people don’t call 911: ending routine police attendance at drug overdoses. Int J Drug Policy.

[40] Townsend T, Blostein F, Doan T, Madson-Olson S, Galecki P, Hutton DW (2020). Cost-effectiveness analysis of alternative naloxone distribution strategies: first responder and lay distribution in the United States. Int J Drug Policy.

[41] Davis CS, Ruiz S, Glynn P, Picariello G, Walley AY (2014). Expanded access to naloxone among firefighters, police officers, and emergency medical technicians in Massachusetts. Am J Public Health.

[42] Wermeling DP (2015). Review of naloxone safety for opioid overdose: practical considerations for new technology and expanded public access. Ther Adv Drug Saf.

[43] Rando J, Broering D, Olson JE, Marco C, Evans SB (2015). Intranasal naloxone administration by police first responders is associated with decreased opioid overdose deaths. Am J Emerg Med.

[44] White MD, Watts S, Orosco C, Perrone D, Malm A (2022). Leveraging body-worn camera footage to better understand opioid overdoses and the impact of police-administered naloxone. Am J Public Health.

[45] Pozo BD (2022). Reducing the iatrogenesis of police overdose response: time is of the essence. Am J Public Health.

[46] Wood CA, Duello A, Horn P (2021). Overdose response training and naloxone distribution among rural first responders. Rural Mental Health.

[47] Reichert J, Lurigio AJ, Weisner L (2019). The administration of naloxone by law enforcement officers: a statewide survey of police chiefs in Illinois. Law Enforc Executive Forum.

[48] Moallef S, Hayashi K (2021). The effectiveness of drug-related Good Samaritan laws: a review of the literature. Int J Drug Policy.

[49] Zadoretzky C, McKnight C, Bramson H, Des Jarlais D, Phillips M, Hammer M, Cala ME (2017). The New York 911 Good Samaritan Law and opioid overdose prevention among people who inject drugs. World Med Health Policy.

[50] Jakubowski A, Kunins HV, Huxley-Reicher Z, Siegler A (2018). Knowledge of the 911 Good Samaritan Law and 911-calling behavior of overdose witnesses. Subst Abuse.

[51] Rouhani S, Schneider KE, Rao A, Urquhart GJ, Morris M, LaSalle L, Sherman SG (2021). Perceived vulnerability to overdose-related arrests among people who use drugs in Maryland. Int J Drug Policy.

[52] Schneider KE, Park JN, Allen ST, Weir BW, Sherman SG (2020). Knowledge of Good Samaritan Laws and beliefs about arrests among persons who inject drugs a year after policy change in Baltimore Maryland. Public Health Rep.

[53] Groh WJ, Newman MM, Beal PE, Fineberg NS, Zipes DP (2001). Limited response to cardiac arrest by police equipped with automated external defibrillators: lack of survival benefit in suburban and rural Indiana–the police as responder automated defibrillation evaluation (PARADE). Acad Emerg Med.

[54] Husain S, Eisenberg M (2013). Police AED programs: a systematic review and meta-analysis. Resuscitation.

[55] Davis CS, Carr D (2015). Legal changes to increase access to naloxone for opioid overdose reversal in the United States. Drug Alcohol Depend.

[56] Dahlem CHG, King L, Anderson G, Marr A, Waddell JE, Scalera M (2017). Beyond rescue: implementation and evaluation of revised naloxone training for law enforcement officers. Public Health Nurs.

[57] Beletsky L, Heller D, Jenness SM, Neaigus A, Gelpi-Acosta C, Hagan H (2014). Syringe access, syringe sharing, and police encounters among people who inject drugs in New York City: a community-level perspective. Int J Drug Policy.

[58] Hill LG, Zagorski CM, Loera LJ (2022). Increasingly powerful opioid antagonists are not necessary. Int J Drug Policy.

[59] Krieter P, Chiang N, Gyaw S, Skolnick P, Crystal R, Keegan F (2016). Pharmacokinetic properties and human use characteristics of an FDA-approved intranasal naloxone product for the treatment of opioid overdose. J Clin Pharmacol.

